# A New Method for the Measurement of the Diffusion Coefficient of Adsorbed Vapors in Thin Zeolite Films, Based on Magnetoelastic Sensors

**DOI:** 10.3390/s20113251

**Published:** 2020-06-07

**Authors:** Dimitris Kouzoudis, Theodoros Baimpos, Georgios Samourgkanidis

**Affiliations:** 1 Department of Chemical Engineering, University of Patras, GR 26504 Patras, Greece; g.samourgkanidis@gmail.com; 2 National Observatory of Athens, Lofos Koufou, P. Pendeli, GR-15236 Athens, Greece; t.baimpos@noa.gr

**Keywords:** magnetoelastic, sensors, diffusion coefficient, zeolite, FAU, LTA

## Abstract

In the current work an experimental method is used in order to calculate the diffusivity D (diffusion coefficient) of various vapors in thin zeolite films. The method is based on adsorption data from magnetoelastic sensors on top of which a zeolite layer was synthesized, and the diffusivity is extracted by fitting the data to Fick’s laws of diffusion. In particular, the method is demonstrated for two volatile organic compound (VOC) vapors on two different zeolites, the p-Xylene adsorption in Faujasite type zeolite with $D=1.89\times 10^{-13}\;m^{2}/s$ at 120 °C and the propene adsorption in Linde Type A type zeolite with $D=5.9\times 10^{-14}\;m^{2}/s$ at 80 °C, two diffusion coefficients which are extracted experimentally for first time. Our results are within the order of magnitude of other VOC/zeolite values reported in literature.

## 1. Introduction

**Figure 1:**
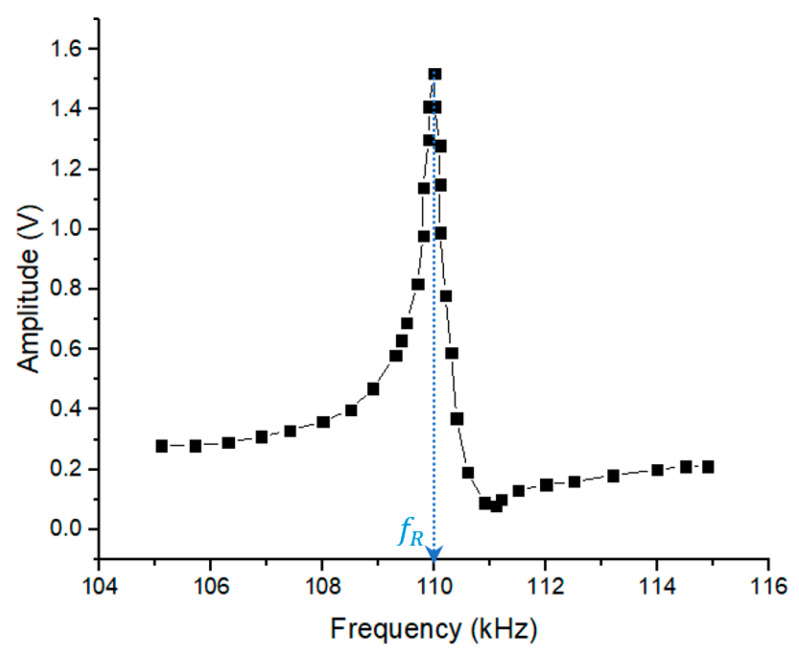
Standard voltage-frequency signal of a magnetoelastic sensor where the resonance peak is clearly seen.

Magnetoelastic sensors are an excellent tool for adsorption studies in thin films as their signal is sensitive to mass loads [1,2,3,4,5,6]. As it is shown in Figure 1, the voltage signal of a thin magnetoelastic strip, when excited along its long axis, exhibits resonance at a specific resonance frequency $f_{R}$. In general, it can be proven that a rectangular thin ribbon with length L of any material with density ρ, Young modulus E, and Poisson’s ratio ν, resonates mechanically [7] at a frequency:

(1) $$f_{R}=\frac{1}{2L}\sqrt{\frac{E}{\rho \left(1-\nu^{2}\right)}}$$

Typically, the magnetoelastic adsorption sensors are composed of two layers, one with the magnetoelastic material which acts as the sensing layer, and one with a chemically active material which selectively adsorbs the desired species. The adsorbed mass alters the density ρ in Equation (1) (because in an adsorption process the sensor mass changes but its volume remains the same) and the resonance frequency shifts toward lower values, assuming that the adsorption does not affect E (there are some exceptions with absorption-induced stresses that alter this assumption which are discussed later in the text). Thus, a proper calibration with known mass loads, can turn the resonating layers into microbalance sensors [1,2,3,4]. Our group has used successfully zeolite films as the chemically active layers, on top of magnetoelastic materials of type “Metglas”, in order to detect a number of different gasses and analytes [5,6].

On the other hand, volatile organic compounds (VOC) are considered hazardous in general and thus their detection and filtration are deemed necessary. Concerning the detection, a number of different detection techniques have been proposed in the past [6,8,9,10,11,12,13,14]. As for filtration, zeolites have played an active role towards this direction, due to their microporous structure, which allows for the trapping and the subsequent removal of VOC from certain atmospheres or other contaminated substances [15,16,17,18]. There are cases though, where removal of VOC by catalytic conversion has been successfully performed [19,20,21].

Of great importance in these applications, is the dynamics of the adsorption of VOC in the zeolite crystals which is basically the diffusion process. When it comes to diffusion, Fick’s two laws come to mind, which can be summarized in the following differential equation,

(2) $$\frac{\partial c}{\partial t}=D\frac{\partial^{2}c}{\partial x^{2}}$$

which governs the diffusion process in one dimension. Here c is the concentration of the adsorbed VOC molecules (mass per distance) and D their diffusion coefficient in the zeolite film. A little discussion about the validity of the above equation should be given here. According to [22], Fick’s laws appear to be valid only for homogeneous systems which is not the case for zeolite crystals when viewed at atomic scale, but they can be considered practically homogeneous when a large space scale is considered. In our case, our zeolite films had thicknesses of the order of micron-meters and the diffusion took place over this distance so we can safely assume that we are on the large scale side. Additionally, our films were not single crystals but were composed of a large number of single crystals (see SEM micrographs in [23]) which means that every physical property of the film has the meaning of an averaged quantity over all possible directions, thus uniformity can be safely assumed. Under these considerations, we can accept Fick’s laws as valid laws to describe diffusion phenomena in our zeolite films.

Another point that needs to be considered is the fact that Equation (2) is valid for the case where only one species is diffusing through a solid material. When two or more species are present, the species do not act independently of each other but the diffusion of one affects the diffusion of the other species [24]. In such a case, the more general Maxwell–Stefan equations need to be used (see Equation (2) in [24]) and Equation (2) above is a special case of these equations when only one species is present. In our case, a binary mixture of gases was used with one being the VOC vapor. However, as the VOC are strongly adsorbed in the zeolite crystal and we are particularly interested on their diffusion dynamics, we can approximately use Equation (2) keeping in mind that the Maxwell–Stefan equations lead only to small corrections to the concentration profiles predicted by Equation (2).

The main purpose of the current work is to use our VOC sensing data which were recorded by magnetoelastic/zeolite strips, in order to extract the diffusion coefficient D in Equation (2) of the VOC in the zeolite crystal. The usefulness of the current method is that (a) there are not so many available VOC-zeolite data for D, (b) when available, there is a noticeable deviation among different authors, and (c) there is a variety of measuring techniques, some of which are not so easy to use and not so direct to interpret. Table 1 below gives a short review of diffusion coefficients D found by different authors for a number of VOC-zeolite combinations.

## 2. Materials and Methods

**Figure 2:**
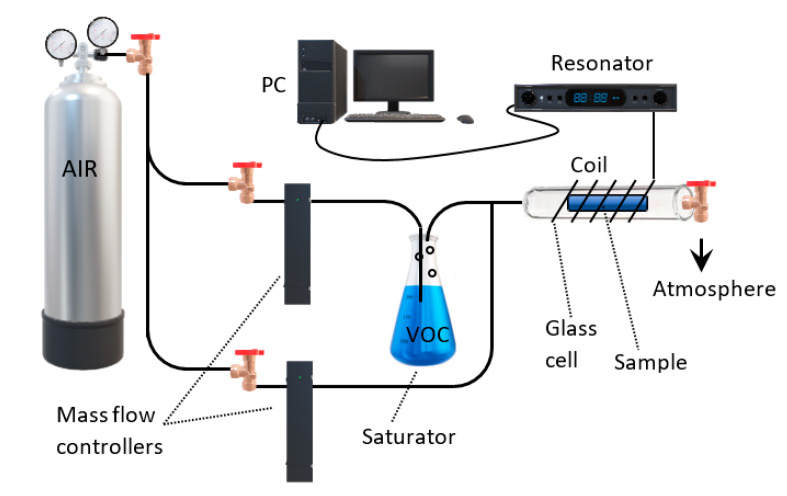
Experimental set-up for the detection of volatile organic compounds (VOC).

**Figure 3:**
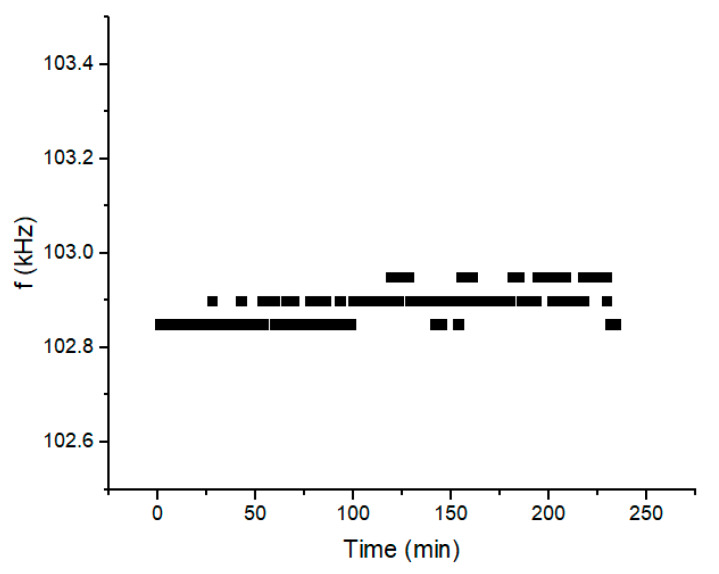
Measurement of the sensor resonance frequency for a period of 4 h under the presence of plain air, to check for the sensor stability.

Figure 2 shows the experimental part that was used for the recording of the VOC adsorption data by magnetoelastic sensors. Our sensors were composed of two layers, one of which was of zeolite type, either Faujasite (FAU) or Linde Type A (LTA) (for more synthesis details and methods, please see [8]) of a few tens of microns thick, and another 30 µm thick layer composed of magnetoelastic material 2826 ΜΒA, which is an amorphous metal with average stoichiometry Fe_40_Ni_38_Mo_4_B_18_. The sensor thickness and width were 2 and 0.4 cm correspondingly. The sensor was placed inside the glass-cell shown in Figure 2 and an excitation coil and a detection coil were wound around the cell. These coils were driven and interrogated by a special resonator which basically resonates the sensor and records its natural frequency on a personal computer. Before allowing any VOC atmosphere in the glass cell, the sensor was left overnight under the flow of dry synthetic air (referred to simply as “air” from now on) at a temperature of 120 or 80 °C (depending on the VOC, see adsorption experiments below) in order to clear its pores of unwanted components such as humidity. Following this clean-up procedure, a number of resonance data were recorded for a total period of 4 h with the same dry air flow and temperature. The results are shown in Figure 3. This experiment proves, as expected, that under a stable atmosphere the resonance frequency f of the laminate remains constant with a mean value of 102.88 kHz and a fluctuation of about ±0.05 kHz, thus providing us with an indication of the accuracy of the set-up. Going back to Figure 2, which was used for the VOC measurements, a saturator was used to create saturated VOC vapor when the VOC was in the liquid phase at normal conditions. As the figure shows, air was allowed to flow through the saturator which contained the liquid VOC. Parallel to that flow, was a dry flow of air and the two flows were mixed in a given ratio in order to create the desired VOC concentration inside the glass cell. The saturator was replaced by a VOC gas cylinder for certain VOC that exist in the gas phase at normal conditions. Two different VOC were tested in the current work by two different sensors: (a) p-Xylene with a sensor with a FAU zeolite, and (b) propene with a sensor with an LTA zeolite. These combinations were chosen because these zeolites show selectivity on these two particular VOC.

## 3. Results

### 3.1. Case (a) p-Xylene/FAU

**Figure 4:**
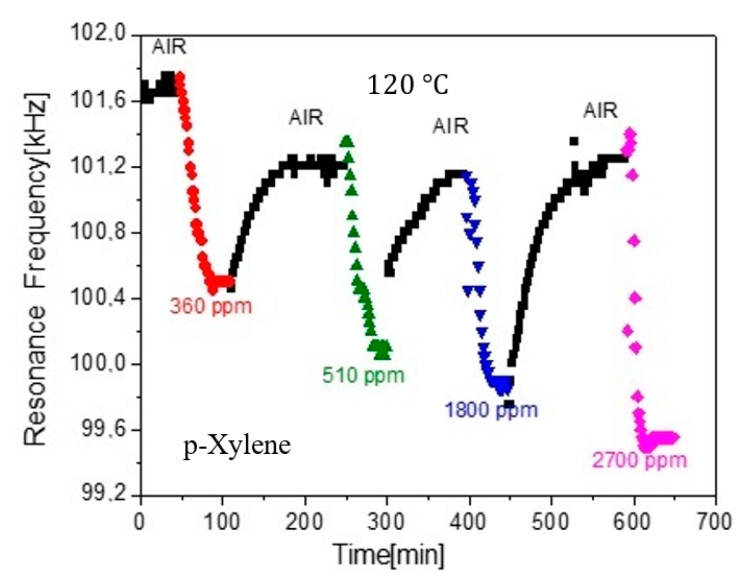
Data from PhD work [35], which corresponds to different p-Xylene concentrations on a Metglas/Faujasite (FAU) sensor.

In this case, the VOC chosen was p-Xylene and the zeolite layer on the sensor was of FAU type and the adsorption measurements at 120 °C were as shown in Figure 4. These measurements are not new, but they were first presented in one of the author’s PhD work [35]. In order to check the zeolite adsorption response, the sensor atmosphere was allowed to alternate between dry-air and VOC of various concentrations in ppm (parts per million with respect to clear air), and several resonance data were recorded in fixed time intervals. The plot shows the corresponding resonance frequencies f versus time. As it was mentioned in the introduction, f depends inversely on mass loads and so it drops when the VOC is introduced in the glass cell, after this was filled with clear air (since p-Xylene is heavier than air). It should be mentioned at this point, that at certain adsorption experiments, there was another important parameter, besides the mass load, that needed to be taken into consideration. This parameter was the development of internal adsorption-stresses on the zeolite film, which were capable of rising f during adsorption, thus contradicting our initial assumptions of a decreasing frequency with mass load. However, in the data of Figure 4, such a behavior was not observed and so we were confident that the measured resonance frequency was entirely due to mass loads.

### 3.2. Case (b) Propene/LTA

**Figure 5:**
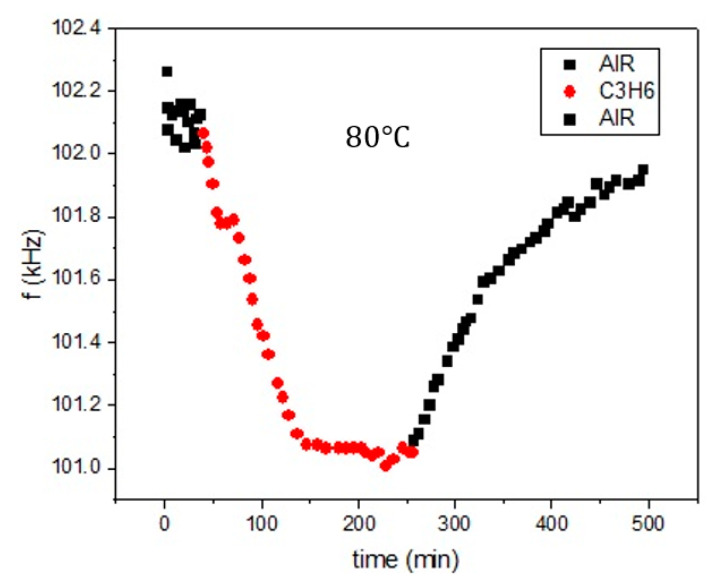
Data from PhD work [35], which corresponds to different propene concentrations on a Metglas/Linde Type A (LTA) sensor.

In this case, the VOC chosen was propene ($C_{3}H_{6}$) and the zeolite layer on the sensor was of LTA type and the adsorption measurements at 80 °C were as shown in Figure 5. As in Case a, these measurements are not new, but they were first presented in the PhD work [35]. Here the sensor atmosphere was subsequently cycled with equal time intervals of 100% air and 100% VOC. Here too, stress-related phenomena were not present in the measurements.

## 4. Discussion

**Figure 6:**
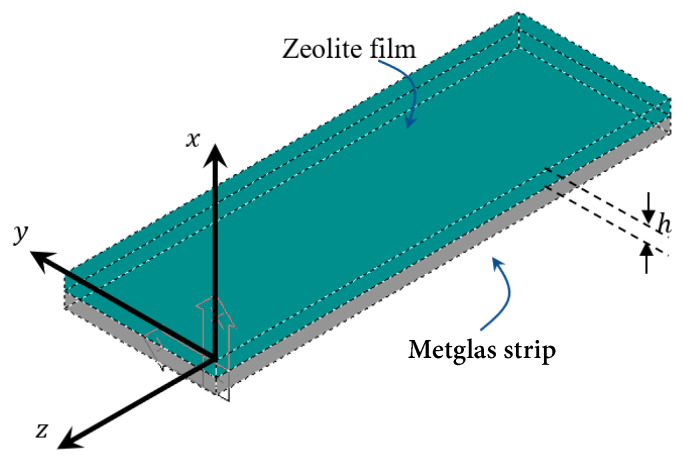
Sensor geometry.

As it was mentioned above, the main purpose of the current paper is to introduce a new method to extract the diffusion constant D of various VOC in Zeolites, given plots like the ones shown in Figure 4 and Figure 5. Our proposed method, consists of the following steps:

- Assume the sensor geometry of Figure 6.
- Solve the differential Equation (2) by applying the appropriate boundary and initial conditions for the VOC concentration c.
- Extract an expression for the VOC adsorbed mass Δm(t) in the Zeolite film versus time. This expression includes the diffusion constant D as a parameter.
- Substitute Δm(t) in Equation (1) to extract a corresponding expression of the sensor resonance frequency f versus time.
- Fit the f expression found in the previous step to the data of Figure 4 and Figure 5 with D as the running parameter and get its optimum value for best fit.

The math details and calculations of the proposed method are explained in detail in the Appendix A, Appendix B, Appendix C. It will only be mentioned here that the fit was applied to only one of the multiple branches of the plots of Figure 4 and Figure 5 and, more specifically, to one of the diffusion-out branches, when the VOC flow at the sensor neighborhood is set to zero (leaving only a flow of synthetic air around the sensor which causes no adsorption according to Figure 2), as this process leads to an easy exponential solution of the differential Equation (2).

Additionally, the basic assumptions of the method need to be given here. As Figure 6 shows, our sensor consists of the zeolite adsorbing film on top of a Metglas strip. The film has the form of a thin slab so most of the gas escapes from the top surface and not sideways as the film thickness is about 30 μm, which is much smaller than the other two dimensions of 4 and 20 mm. Thus, the gas concentration in the film, will be considered to be constant over *y* and *z* (except only for a small fraction at the edges) and have a strong *x* profile along the film’s thickness. That makes the problem one-dimensional which simplifies the whole analysis.

The analysis in the Appendix B leads to the following Equation (A11) for the sensor resonance frequency f versus time

(3) $$f\approx f_{0}\left[1-\frac{4hc_{0}}{m\pi^{2}}\exp\left(-\frac{D\pi^{2}}{4h^{2}}t\right)\right]$$

where $f_{0}$ is the resonance frequency without mass load (basically with air flow as in Figure 2), h is the zeolite film thickness, $c_{0}$ is the initial VOC concentration in units g/μm inside the film (from the previous diffusion-in process, where $c_{0}$ is assumed to be constant over the film thickness), and m is the clear sensor mass without adsorption. Equation (3) is a simple exponential expression of the form $f=f_{0}\left(1-ae^{-bt}\right)$ with

(4) $$a=\frac{4hc_{0}}{m\pi^{2}}$$

and

(5) $$b=\frac{D\pi^{2}}{4h^{2}}$$

so it can easily be fit to get a and b, in order to extract D from the simple expression

(6) $$D=\frac{4h^{2}b}{\pi^{2}}$$

**Figure 7:**
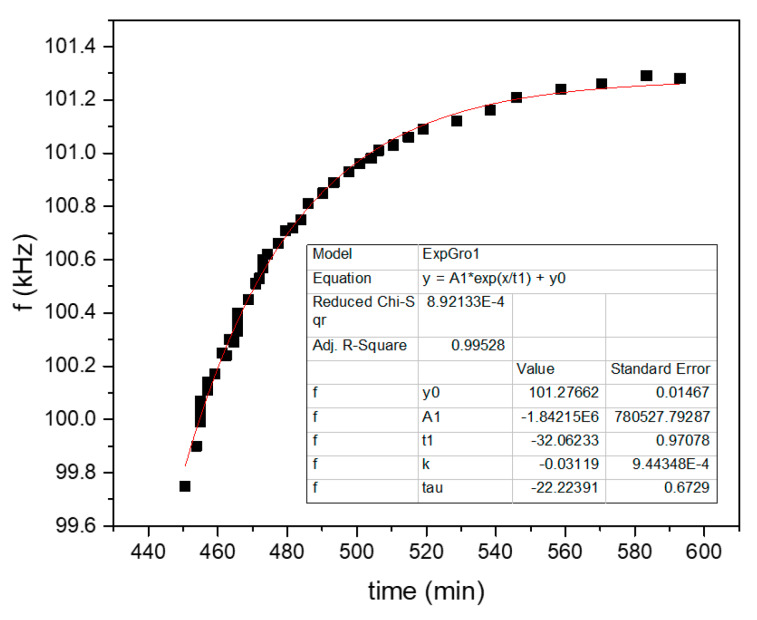
Exponential fit of the second branch marked “AIR” at the plot of Figure 4.

**Figure 8:**
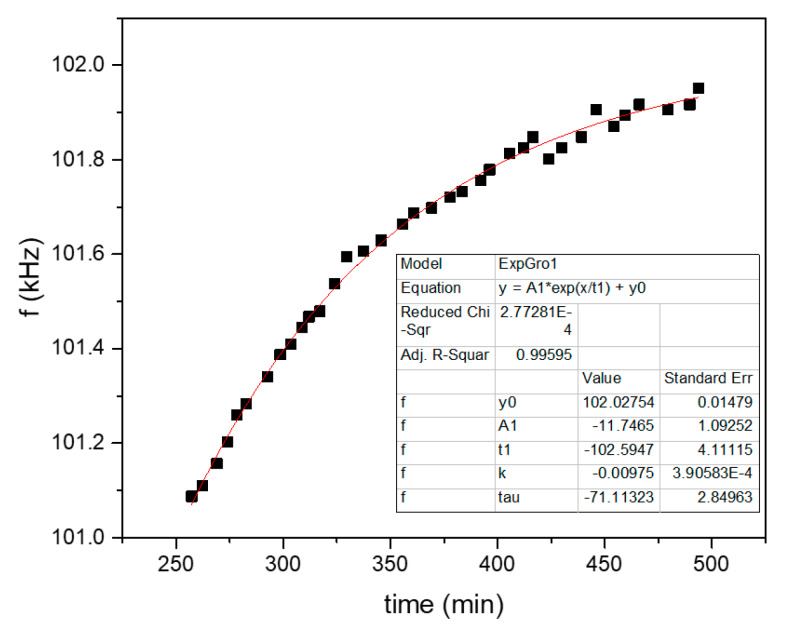
Exponential fit of the second branch marked “AIR” at the plot of Figure 5.

Shown in Figure 7 and Figure 8 are the fits of the plots of Figure 4 and Figure 5, correspondingly, of only the first diffuse-out branch (the second marked “AIR” branch in the plots). The corresponding fit parameters and diffusion coefficients are shown in Table 2.

Thus the mini review list of Table 1 in Section 1, can be enriched by adding two extra lines for the cases of p-Xylene in FAU type zeolite and propene in LTA type zeolite of the current work, with corresponding diffusion coefficients D of $1.89\times 10^{-13}\;m^{2}/s$ at 120 °C and $5.9\times 10^{-14}\;m^{2}/s$ at 80 °C, correspondingly. Both results are within the order of magnitude of the values presented in the table.

## 5. Conclusions

A new experimental technique was proposed which is able to determine the diffusion coefficient of a gas in a porous adsorbing medium such as a zeolite, but it can equally being applied to other porous materials. The technique is based on the use of resonance data from magnetoelastic sensors, enhanced by an exponential data fit model of Fickian type diffusion. The method is demonstrated for two different cases, the adsorption of p-Xylene vapor in a FAU zeolite and propene gas in an LTA zeolite and the corresponding diffusion coefficients D of $1.89\times 10^{-13}\;m^{2}/s$ at 120 °C and 

$5.9\times 10^{-14}\;m^{2}/s$ at 80 °C, which are reported here for first time and are within the order of magnitude of results found for other pairs in the literature. The importance of the current work for future study is that more pairs of VOC/zeolite diffusion coefficients can be easily measured as there are not so many data available in the literature, thus enriching Table 1 and providing a good reference for research in the field.

## Figures and Tables

**Figure A1:**
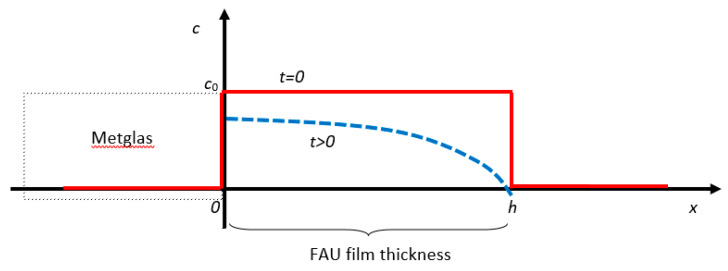
Initial and later gas concentration across the film thickness.

**Table 1:** Different diffusion coefficients for gas/zeolite pairs reported in the literature.

VOC	Zeolite Type	$D\;\left(10^{-12}\;m^{2}/s\right)$	Temperature (°C)	Reference
p-Xylene	ZSM-5	0.13	300	[25]
Benzene	ZSM-5	0.025	65	[26]
Benzene	ZSM-5	0.05	142	[27]
ethylbenzene	ZSM-5	0.049	142	[27]
i-Butane	ZSM-5	1	60	[28]
n-Butane	ZSM-5	0.8	200	[29]
n-Hexane	ZSM-5	0.46	200	[29]
2-Methylpentane	MFI	1	130	[30]
n-Hexane	MFI	45	130	[30]
methanol	NaX	10	100	[27]
Benzene	NaX	12	-	[27]
Benzene	NaX	12	195	[31]
propylene	NaY	1500	-	[32]
p-Xylene	NaY	0.18	25	[33]
Propene	5A	1.1–1.6	200	[29]
n-Butane	5A	0.5–0.7	200	[29]
n-Octane	USY	1100	25	[22]
n-Butane	Modernite	24,000	60	[28]
n-Hexane	Pt/HMOR	0.1	250	[34]

**Table 2:** Fit results of Figure 7 and Figure 8.

	h (μm)	$b\;\left(min^{-1}\right)$	$D\;\left(m^{2}/s\right)$	θ (°C)
FAU/p-Xylene	30	0.0312	$1.89\times 10^{-13}$	120
LTA/propene	30	0.00975	$5.9\times 10^{-14}$	80

## Appendix A. Mathematics of Diffusion

In what follows, we will follow the analysis in [36]. The basic equation that governs the diffusion process in one dimension is

(A1) $$\frac{\partial c}{\partial t}=D\frac{\partial^{2}c}{\partial x^{2}}$$

where c is the concentration of the adsorbed gas molecules (mass per distance for problems in one dimension) and D their diffusion coefficient in the host zeolite. As shown in Figure A1 below, our zeolite film of thickens h is confined between the plane x=0, which is the zeolite-sensor interface, and x=h which is the open surface. The other two film dimensions y and z are considered to be infinite. It is assumed that the film was exposed for long time to a steady gas flow which brings the film to a saturated state with a constant concentration $c=c_{0}$ all over the film. That will be the initial condition at t=0. Then, suddenly, the gas flow is interrupted, and the gas is slowly diffusing out from the free end at x=h where the concentration is considered to be zero, which means c(h,t)=0 for all times t>0. As the diffusion progresses, a certain amount of gas is still present in the film and we would like to know the concentration profile so as to calculate the remaining gas mass. Another boundary condition is at the x=0 surface where the gas cannot escape out and thus its flow there is zero. This mathematically is expressed by a flat curve at this edge, in other words ∂c(0,t)/∂x=0 for all times t>0. We expect the concentration to have a general form like the one shown with a blue dashed line in the figure.

In Section 2.3 of [36], the author gives a general solution of the diffusion Equation (A1) by the method of separation of variables. The solution has the form (Equation 2.24 in the Reference [36])

(A2) $$c=\left(A\sin\lambda x+B\cos\lambda x\right)\exp\left(-D\lambda^{2}t\right)$$

From the second boundary condition ∂c(0,t)/∂x=0, we have necessarily A=0. From the other boundary condition c(h,t)=0, we have

$$\cos\lambda h=0=>\lambda h=\left(2n+1\right)\frac{\pi }{2}$$

or

(A3) $$\lambda_{n}=\left(2n+1\right)\frac{\pi }{2h}$$

where n=0,1,2,…. Since for each n we have a different solution in Equation (A2), the most general solution is the sum of all solutions (because the differential Equation (A1) is linear in c). Summing all terms, we get

(A4) $$c\left(x,t\right)=\overset{\infty }{\underset{n=0}{\sum }}B_{n}\cos\left(\lambda_{n}x\right)\exp\left[-D\lambda_{n}{}^{2}t\right]$$

where $\lambda_{n}$ is given by Equation (A3). From the initial condition at t=0 we have for each x in the film

(A5) $$c_{0}=\overset{\infty }{\underset{n=0}{\sum }}B_{n}\cos\left(\lambda_{n}x\right)$$

We can use this relationship to find $B_{n}$ with the help of the cosine orthogonality rule according to which:

(A6) $$\int_{0}^{h}\cos\lambda_{n}x\cos\lambda_{m}xdx=\frac{h}{2}\delta_{mn}$$

To make use of this rule, we multiply Equation (5) by $\cos\left(\lambda_{m}x\right)$ and integrate from x=0 to x=h:

$$\int_{0}^{h}c_{0}\cos\left(\lambda_{m}x\right)dx=\overset{\infty }{\underset{n=0}{\sum }}B_{n}\int_{0}^{h}\cos\left(\lambda_{m}x\right)\cos\left(\lambda_{n}x\right)dx$$

or

$$c_{0}\frac{\sin\left(\lambda_{m}h\right)}{\lambda_{m}}=\frac{h}{2}\overset{\infty }{\underset{n=0}{\sum }}B_{n}\delta_{mn}=>\frac{c_{0}}{\lambda_{m}}\sin\left[\left(2m+1\right)\frac{\pi }{2}\right]=\frac{h}{2}B_{m}$$

In the last step, use was made of Equations (A3) and (A6). The sine term evaluates at even multiples of π/2 so it is either 1 or −1, depending on the value of m. Therefore,

$$B_{m}=\frac{2c_{0}}{h\lambda_{m}}{\left(-1\right)}^{m}=\frac{4c_{0}}{\left(2m+1\right)\pi }{\left(-1\right)}^{m}$$

Equation (A4) then becomes

(A7) $$c=\overset{\infty }{\underset{n=0}{\sum }}\frac{4c_{0}}{\left(2n+1\right)\pi }{\left(-1\right)}^{n}\exp\left[-D\lambda_{n}{}^{2}t\right]\cos\left(\lambda_{n}x\right)$$

For increasing values of n, the factor ${\left(2n+1\right)}^{2}$ becomes increasingly larger, like 1, 9, 25, 49,… etc., and it appears both in the denominator and the exponent of each term in the sum. Thus, successive terms in the sum are by far smaller than the previous terms. That means that it is a good approximation to keep only the n=0 term in Equation (A7) and have

(A8) $$c=\frac{4}{\pi }c_{0}\exp\left[-D\lambda_{0}{}^{2}t\right]\cos\left(\lambda_{0}x\right)$$

where $\lambda_{0}=\pi /2h$ (Equation (A4)). To find the total mass Δm(t) that remains in the film at time t (part of the mass diffuses out of the film), we need to integrate Equation (A8) from x=0 to x=h. Since

$$\int_{0}^{h}\cos\left(\lambda_{0}x\right)dx=\frac{\sin\left(\lambda_{0}h\right)}{\lambda_{0}}=\frac{2h}{\pi }\sin\frac{\pi }{2}=\frac{2h}{\pi }$$

we have

$$\Delta m\left(t\right)=\int_{o}^{h}cdx=\frac{8h}{\pi^{2}}c_{0}\exp\left[-D\lambda_{0}{}^{2}t\right]$$

The limiting values of the mass are Δm(∞)→0 for t→∞ as expected, and the initial value

$$\Delta m\left(0\right)=\frac{8h}{\pi^{2}}c_{0}$$

for t=0.

## Appendix B. Connection with the Resonance Frequency

The density ρ of the sensor when no gas is present is ρ=m/V which changes under adsorption to:

(A9) $$\rho =\frac{m+\Delta m}{V}$$

where m is the sensor’s mass in vacuum (with no adsorption), V its volume, and Δm the amount of the adsorbed mass. From Equation (1) in the main context of the current paper, we have:

$$f=\frac{1}{2L}\sqrt{\frac{E}{\rho }}$$

where E is the sensor Young’s modulus and L its length. With the help of Equation (A9) this becomes

(A10) $$f=\frac{1}{2L}\sqrt{\frac{VE}{m+\Delta m}}$$

Typically, the mass load Δm is but a tiny percent of the total mass m of the sensor which means that Δm/m ≪ 1. Using the approximation ${\left(1+x\right)}^{n}\approx 1+nx$ for small x, we have

$$f=\frac{1}{2L}\sqrt{\frac{VE}{m+\Delta m}}=\frac{1}{2L}\sqrt{\frac{VE}{m\left(1+\Delta m/m\right)}}\approx f_{0}\left(1-\frac{1}{2}\frac{\Delta m}{m}\right)$$

where $f_{0}$ is the resonance frequency without mass load (the f of Equation (A10) for Δm=0). With the help of Equation (A9) the above expression becomes:

(A11) $$f\approx f_{0}\left[1-\frac{4h}{m\pi^{2}}c_{0}\exp\left(-\frac{D\pi^{2}}{4h^{2}}t\right)\right]$$

where the value $\lambda_{0}=\pi /2h$ from Equation (3) was used in the exponent. The above equation has a simple exponential form

$$f=f_{0}\left(1-ae^{-bt}\right)$$

where

(A12) $$a=\frac{4hc_{0}}{m\pi^{2}}$$

and

(A13) $$b=\frac{D\pi^{2}}{4h^{2}}$$

## Appendix C. Orthogonality Condition

Proof of orthogonality of $\cos\lambda_{n}x$ functions: from basic trigonometry we have

$$2\cos\lambda_{n}x\;\cos\lambda_{m}x=\cos\left(\lambda_{n}+\lambda_{m}\right)x+\cos\left(\lambda_{n}-\lambda_{m}\right)x$$

Consider two different cases:

(a) Case m≠n

$$\begin{array}{c} 2\int_{0}^{h}\cos\lambda_{n}x\cos\lambda_{m}xdx=\int_{0}^{h}\cos\left(\lambda_{n}+\lambda_{m}\right)xdx+\int_{0}^{h}\cos\left(\lambda_{n}-\lambda_{m}\right)dx\\ =\frac{1}{\left(\lambda_{n}+\lambda_{m}\right)}{\left[\sin\left(\lambda_{n}+\lambda_{m}\right)x\right]}_{0}^{h}+\frac{1}{\left(\lambda_{n}-\lambda_{m}\right)}{\left[\sin\left(\lambda_{n}-\lambda_{m}\right)x\right]}_{0}^{h}\\ =\frac{1}{\left(\lambda_{n}+\lambda_{m}\right)}\sin\left(\lambda_{n}+\lambda_{m}\right)h+\frac{1}{\left(\lambda_{n}-\lambda_{m}\right)}\sin\left(\lambda_{n}-\lambda_{m}\right)h\\ =\frac{1}{\left(\lambda_{n}+\lambda_{m}\right)}\sin\left(2n+2m+2\right)\frac{\pi }{2}+\frac{1}{\left(\lambda_{n}-\lambda_{m}\right)}\sin\left(2n-2m\right)\frac{\pi }{2} \end{array}$$

In both expressions, the argument of the sine function is an even integer times π/2 which evaluates to either π or 2π (in the first trigonometric cycle) where the sine is zero. Therefore, when n differs from m we have:

$$\int_{0}^{h}\cos\lambda_{n}x\cos\lambda_{m}xdx=0$$

(b) Case m=n

We have

$$\begin{array}{ll} 2\int_{0}^{h}\cos\lambda_{n}x\cos\lambda_{n}xdx & =\int_{0}^{h}\cos\left(2\lambda_{n}\right)xdx+\int_{0}^{h}\cos\left(0\right)dx\\ & =\frac{1}{2\lambda_{n}}{\left[\sin\left(2\lambda_{n}\right)x\right]}_{0}^{h}+\int_{0}^{h}dx=\frac{1}{2\lambda_{n}}\sin\left(2\lambda_{n}h\right)+h\\ & =\frac{1}{2\lambda_{n}}\sin\left(2n+1\right)\pi +h=h \end{array}$$

Concluding the two cases, we can write this in one formula:

$$\int_{0}^{h}\cos\lambda_{n}x\cos\lambda_{m}xdx=\frac{h}{2}\delta_{mn}$$
